# Recombinant Measles AIK-C Vaccine Strain Expressing the prM-E Antigen of Japanese Encephalitis Virus

**DOI:** 10.1371/journal.pone.0150213

**Published:** 2016-03-01

**Authors:** Akira Higuchi, Hiroko Toriniwa, Tomoyoshi Komiya, Tetsuo Nakayama

**Affiliations:** 1 Kitasato-Daiichi Sankyo Vaccine, Division of Vaccine Production, Kitamoto City, Saitama Prefecture, 364–0026, Japan; 2 Kitasato-Daiichi Sankyo Vaccine, Division of Vaccine Development, Kitamoto City, Saitama Prefecture, 364–0026, Japan; 3 Kitasato Institute of Life Sciences, Laboratory of Viral Infection, Tokyo, 108–8641, Japan; University of Georgia, UNITED STATES

## Abstract

An inactivated Japanese encephalitis virus (JEV) vaccine, which induces neutralizing antibodies, has been used for many years in Japan. In the present study, the JEV prM-E protein gene was cloned, inserted at the P/M junction of measles AIK-C cDNA, and an infectious virus was recovered. The JEV E protein was expressed in B95a cells infected with the recombinant virus. Cotton rats were inoculated with recombinant virus. Measles PA antibodies were detected three weeks after immunization. Neutralizing antibodies against JEV developed one week after inoculation, and EIA antibodies were detected three weeks after immunization. The measles AIK-C-based recombinant virus simultaneously induced measles and JEV immune responses, and may be a candidate for infant vaccines. Therefore, the present strategy of recombinant viruses based on a measles vaccine vector would be applicable to the platform for vaccine development.

## Introduction

*Japanese encephalitis virus* (JEV) is a member of the *Flaviviridae* family that is transmitted through the bites of *Culex tritaeniorhynchus* infected with JEV, and is a potential cause of acute encephalitis in humans. Swine are amplifier animals of JEV, while humans are infected as the terminal host through mosquito bites [1]. Acute encephalitis develops in one out of approximately 250 infected cases after a 2-week incubation period, and is characterized by a high fever and non-specific illness, followed by convulsion, headache, confusion, and paralytic illness. JE is still a life-threating disease with severe sequelae [2]. A total of 67,900 clinical cases have been estimated to occur annually, with approximately 20,000 deaths being reported in high risk countries in South East Asia [3]. Approximately 2,000 deaths caused by JEV infection were reported in Japan 1950, however, this number was reduced by the introduction of an inactivated JEV vaccine for children <15 years of age. Several cases of acute encephalitis caused by JEV infection have recently been reported annually, mostly in the elderly, in Japan [4]. Although the incidence of clinical encephalitis is rare, an estimated 3 billion individuals are at risk of JE in high risk countries. No specific therapeutic agent is currently available and prophylactic vaccines are the only effective method to control JE [1].

In Japan, severe cases of acute disseminated encephalomyelitis (ADEM) have been reported following JEV vaccines. The government decided to discontinue mouse brain-derived vaccines because of the potential risk of allergic neurological illnesses. Although mouse brain-derived inactivated vaccines were initially safe in clinical settings, the WHO announced it would be better to replace them with Vero cell culture-based JEV vaccines [5]. In 2010, Vero cell-derived vaccines were introduced in Japan for domestic use [6, 7]. The Vero cell-derived alum adjuvanted vaccine (SA 14-14-2 strain) is now used in most Asian countries, and induces neutralizing antibodies, similar to mouse brain-derived JEV vaccines [8].

Cell-based inactivated vaccines are now widely used in high risk areas and as traveler vaccines, and another vaccine formulation is a live attenuated vaccine. The live attenuated JEV vaccine, based on JEV strain SA 14-14-2 strain, was developed in China using conventional establishment in primary hamster kidney cells and additional passages in embryogenic chick cells, and is now widely used in China and other countries [9]. Through recent development of DNA engineering, chimeric Flavivirus vaccines based on yellow fever vaccine 17D, a well-known ChimeriVax platform, have been applied to *Japanese encephalitis*, *Dengue*, *and West Nile viruses* [10, 11]. The genome region of JEV prM-E in the 17D strain was replaced with that of the JEV SA 14-14-2 strain [10]. This chimeric JEV vaccine (IMOJEV) was shown to induce sufficient immunogenicity, similar to Vero-cell derived inactivated vaccines, in clinical trials, with the most attractive benefit being its efficacy after a single dose immunization [12].

Recombinant measles virus AIK-C vaccine strain expressing the fusion (F), glycoprotein (G), nucleoprotein (NP), and membrane (M)-2 proteins of respiratory syncytial virus (RSV) were constructed in our laboratory. The recombinant AIK-C stain expressing the RSV F or G protein induced neutralizing antibodies, while those expressing the F, NP, or M-2 protein induced cell-mediated immunity, thereby providing protective immunity [13, 14]. Following this line of AIK-C measles vaccine vector, a recombinant measles virus expressing the JEV antigen was investigated, using cotton rats because they are only small experimental animals susceptive to measles virus as shown in previous reports [13, 14]. The prM protein is cleaved to the M protein during virus maturation, to create a structural protein, while the E protein is a major protective envelop antigen that induces neutralizing (NT) and hemagglutination inhibition (HI) antibodies, and the non-structure (NS)-1 protein is thought to be related to replication activity and JEV maturation [15, 16, 17]. The prM-E region is considered to be related to protective antigens and, in the present study, was inserted at the P/M junction of AIK-C cDNA. The characteristics of infectious virus were the investigated for the JEV vaccine candidate.

## Materials and Methods

### 2.1. Virus and cells

JEV Beijing-1 and AIK-C measles vaccine seed stocked strains (Kitasato Daiichi Sankyo Vaccine: Kitamoto, Saitama prefecture, Japan) were used for this work. Vero CCL-81 cells purchased from ATCC were maintained in VP-SFM supplemented with 4 mM L-glutamine. B95a cells were maintained in RPMI-1640 medium supplemented with 10% FBS. 293T cells were maintained in MEM supplemented with 5% FBS, and B95a cells in RPMI 1640 supplemented with10% FCS. BHK cells were maintained in MEM supplemented with 5% FBS for an analysis of the development of NT antibodies against JEV. All media were supplemented with 10,000 IU/ml penicillin and 10,000 μg/ml streptomycin, and irradiated FBS was used. All cell lines were maintained within 50–100 passages and validated in the quality control units of Kitasato Daiichi-Sankyo Vaccine.

### 2.2. Construction of cDNA expressing the JEV prM-E antigen

**Fig 1:**
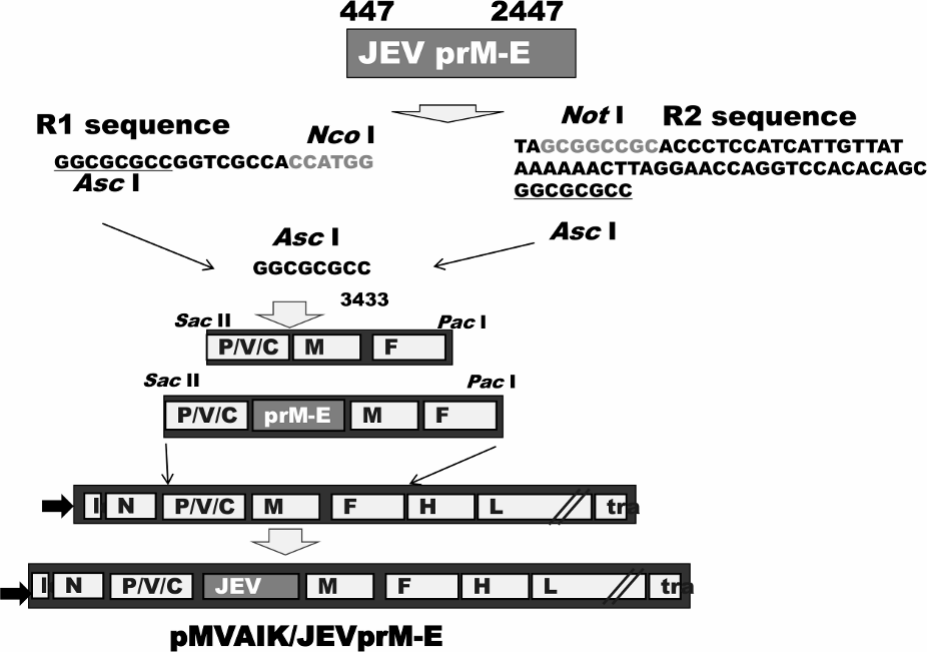
Construction strategy of recombinant cDNA. The PrM-E region was amplified from the Beijin strain of Japanese encephalitis virus and cloned into the P/M junction of pMVAIK, using *Nco* I and *Not* I restriction enzyme sites.

Construction strategy is shown in Fig 1. The AIK-C cloning vector for the foreign genome was previously constructed from the *Sac* II (genome position 2040 of AIK-C) to *Pac* I (genome position 7238 of AIK-C) sites, and the GGCGCG sequence was added upstream of CC of genome position 3433, introducing a new *Asc* I restriction site. Restriction sites R1 linked with *Nco* I and R2 linked with *Not* I sequences were subsequently added [13, 14]. The JEV prM-E gene was cloned from nt position 447 to 2447 of the JEV genome, which had been amplified by PCR using linker primers having *Nco* I and *Not* I sites from the plasmid reported by Hashimoto et al. [18]. The JEV prM-E gene was then inserted at the P/M junction of the cDNA of the AIK-C strain and the full-length cDNA, pMVAIK/JEVprM-E, was constructed.

### 2.3. Recovery of infectious virus

293T cells were infected with the non-replicative recombinant vaccinia virus, MVA T7 Pol, expressing T7 RNA polymerase, and pMVAIK/JEVprM-E was transfected together with TransIT-LT1 Reagent (Mirus Bio Corporation, US) and helper plasmids encoding the Nucleo (N), Phospho (P), and Large (L) proteins of AIK-C. After being incubated for 3 h, the medium was replaced with fresh MEM supplied with 5% FBS. 293T cells were detached after a 2-day culture, and the co-cultured with B95a cells [13, 14].

### 2.4. Virus growth

In order to examine the viral growth, B95a cells were infected with MVAIK/JEVprM-E (m.o.i. = 0.02), and plates were incubated at temperatures of 33, 35, 37, and 39°C. Culture fluids were obtained on days 1, 3, 5, and 7 and infective titers were determined. Infective titers were expressed as TCID50/ml.

### 2.5. Expression of the E protein of JEV

B95a cells were infected with MVAIK/JEVprM-E, and cells were fixed with ethanol. Monoclonal antibodies against the JEV E and measles N proteins were used, and cells were stained using anti-mouse polyclonal antibodies conjugated with FITC or rodamine.

Vero cells were infected with MVAIK/JEVprM-E, and culture supernatants, infected cells, and cell lysates were immunoprecipitated using a polyclonal antibody against JEV raised in rabbits. Proteins were electrophoresed, transferred, and stained using monoclonal antibodies.

### 2.6. Immunogenicity in cotton rats

Seven week-old female cotton rats were purchased from Harlan Laboratories, USA, and were infected with recombinant virus MVAIK/JEVprM-E through intra-muscular route and serum samples were obtained to examine the development of antibodies against measles virus and JEV. Measles PA antibodies were assayed using a PA antibody kit (Fuji revio, Tokyo). EIA and neutralizing antibodies against JEV were examined using the Beijing strain, as previously reported [19, 20]. The study design was approved by the Committee of Animal Research of Kitasato Institute for Life Sciences (Approval Number; 13–003).

During experimental procedures, vaccination, and blood sampling, animals were anesthetized with 3% isoflurane with oxygen. After the research, animals were deeply anesthetized additionally with pentobarbital into peritoneal cavity and necropsied. In the protocol of experimental research, clinical signs to determine when to euthanize the animals were loss of appetite and loss of activities. No animal died during the course of the experiments until they were euthanized.

## Results

### 3.1. Growth of recombinant MVAIK/JEVprM-E virus in B95a cells

**Fig 2:**
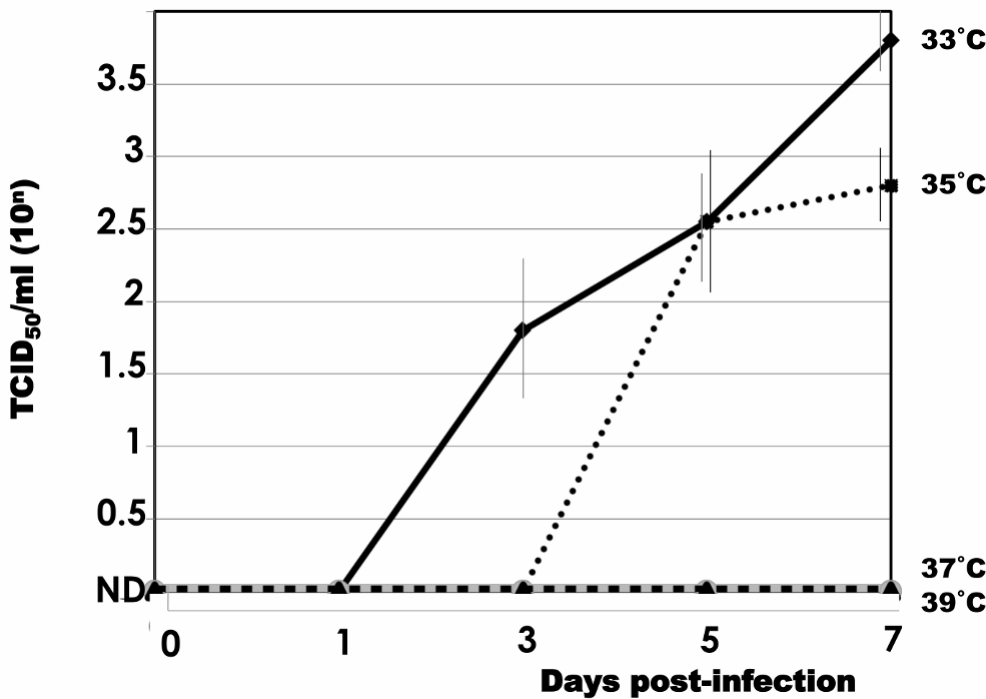
Virus growth in Vero cells at different temperatures of 33, 35, 37, and 39°C. Virus infectivity was assayed as TCID_50_ in Vero cells and each bar represents the mean ± 1.0 SD.

B95a cells were infected with MVAIK/JEVprM-E with moi = 0.02 at different temperatures. Culture supernatants were harvested and the results of infectivity are shown in Fig 2. The virus grew well at 33°C three days after the inoculation, with a peak being on day 7 of the infection. Virus growth was reduced at 35°C and absent at 37°C and 39°C. The recombinant MVAIK/JEVprM-E strain maintained the ts characteristics, as demonstrated in the original measles AIK-C [21].

### 3.2. Expression of the JEV protein

**Fig 3:**
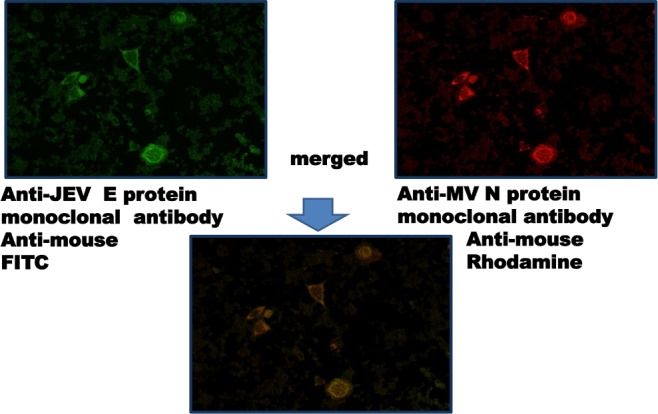
Expression of JEV E and measles N proteins. Monoclonal antibodies against the JEV E and measles N proteins were used, and visualized by anti-mouse antibodies conjugated with FITC or rhodamine.

B95a cells were infected with MVAIK/JEVprM-E and fixed. The results are shown in Fig 3. Cells were stained with monoclonal antibodies against the JEV E and measles N proteins. The JEV E protein was expressed at the same location as the expression of measles N protein.

**Fig 4:**
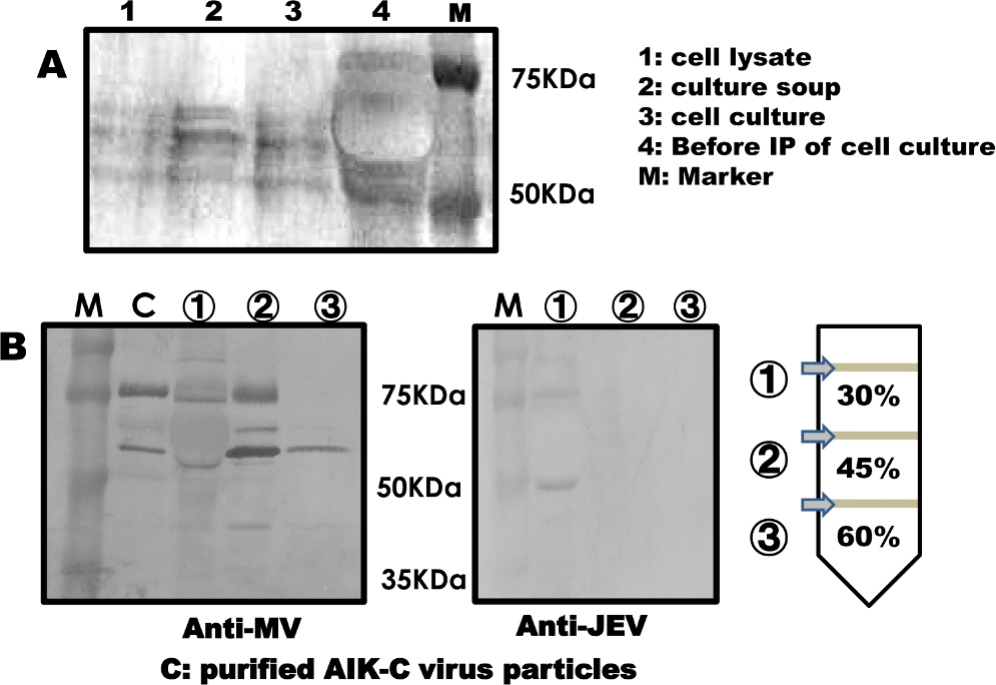
Protein expression of JEV of culture fluid and purified measles virus particles. Vero cells were infected with a recombinant virus, and cell lysates, culture supernatants, and cell culture fluids were then immunoprecipitated using a polyclonal antibody against JEV. They were subjected to Western blotting (4A). Recombinant virus particles were purified through sucrose discontinuous density gradients. Fractions 1, 2, and 3 were collected and subjected the Western blotting. Polyclonal antibodies against measles virus (left panel) and monoclonal antibodies against the JEV E protein were used (4B).

The infected B95a cell lysate, culture supernatant, and cell culture were immuno- precipitated using polyclonal antibodies against JEV and subjected to Western blotting. They were stained with a monoclonal antibody against the JEV E protein, and the results are shown in Fig 4A. Three major bands were from 50–75 KD. The molecular size of the JEV E protein was estimated to be approximately 50K, similar to previously findings reported in expression systems [16, 22].

Vero cells were infected with MVAIK/JEVprM-E and the culture supernatant was collected. It was ultra-centrifuged through 60%, 45%, and 30% sucrose discontinuous gradients. Three fractions were collected and Western blotting was performed using polyclonal antibodies against measles virus and JEV, as shown in Fig 4B. Measles virus was detected in fraction 2. JEV proteins were detected in fraction 1 (supernatant). Fraction 3 contained residual measles particles, but the JEV E protein was not detected in fraction 2 or 3. This result suggested that the JEV E protein was not incorporated into the particles.

### 3.3. Immune responses of the recombinant virus in cotton rats

**Fig 5:**
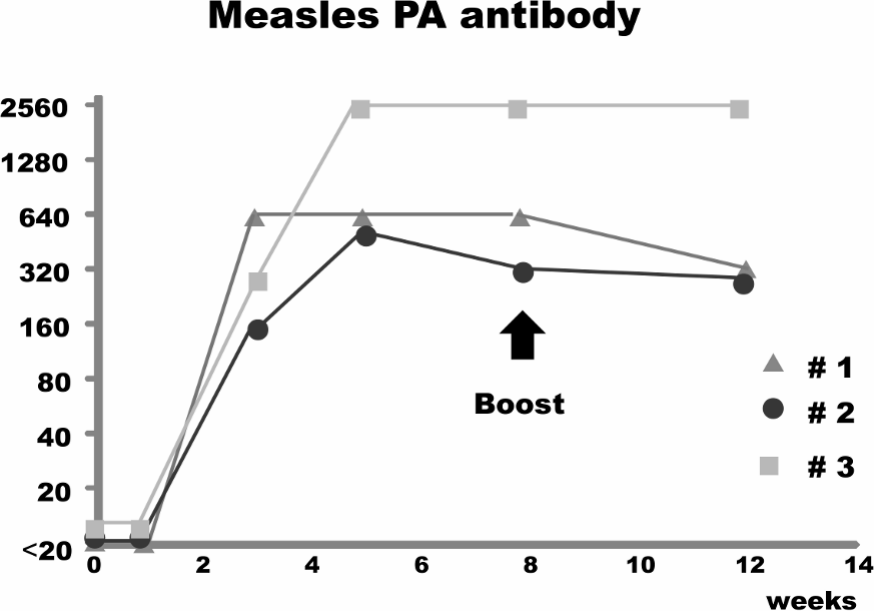
Antibody response against measles virus. Three cotton rats were immunized with a recombinant virus and reimmunized eight weeks after the first immunization. Serum samples were obtained after 1, 3, 5, 8, and 12 weeks. Measles antibodies were examined using a PA antibody kit.

Three cotton rats were immunized with MVAIK/JEVprM-E and re-immunized eight weeks after the first dose. The development of PA antibodies to measles virus is shown in Fig 5. PA antibodies developed three weeks after immunization and peaked at five weeks after immunization. No booster response was noted after re-immunization.

**Fig 6:**
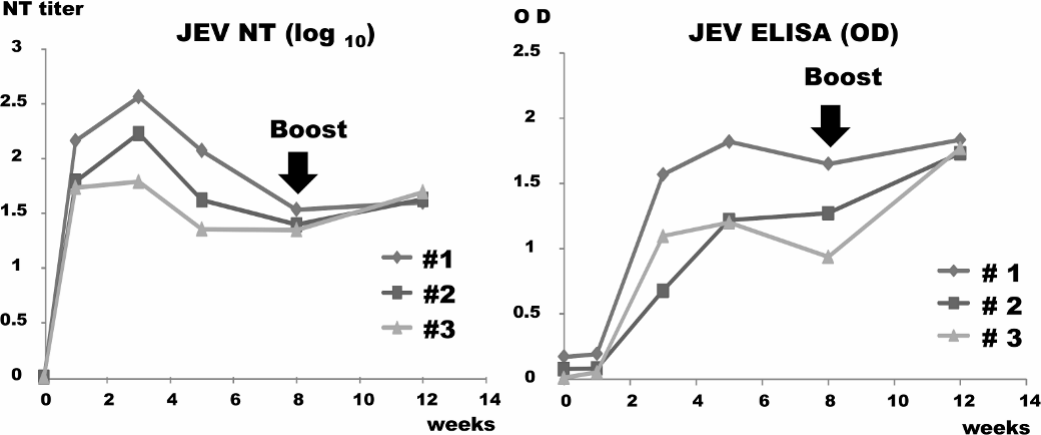
Antibody responses of EIA and NT antibodies against JEV. Three cotton rats were immunized with a recombinant MVAIK/JEVprM-E virus and reimmunized eight weeks after the first immunization. Serum samples were obtained after 1, 3, 5, 8, and 12 weeks. EIA and NT antibodies against JEV were measured.

EIA and neutralizing (NT) antibodies were examined using the Beijing strain, and the results are shown in Fig 6. EIA antibodies developed three weeks after and NT antibodies one week after immunization. No booster response was demonstrated. NT antibodies against JEV were induced three weeks after immunization. Enhanced immune response was noted in two out of three rats but was not significant; one developed a high titer of 10 ^1.65^ six weeks after immunization without antibody response.

## Discussion

JEV is still prevalent in South-East Asia and South Asia. The number of reported cases markedly decreased to less than 10 patients in one year recently in Japan [23]. Antibodies and CTL are both considered to protect against JEV infection based on the findings of animal experiments using the artificial transfer of sera and CTL. Among several JEV proteins, the E protein is regarded as the protective antigen target [16, 17]. Therefore, an inactivated JEV vaccine was developed and recommended for routine immunization. Mouse brain-based vaccines were initially used, but cell-culture based vaccines were subsequently developed [1, 6, 7, 8]. Vero cell-based JEV-inactivated vaccines were shown to induce cross-reactive protective antibodies against *Murray Valley encephalitis virus* and *West Nile virus*, with a delta inulin-based adjuvant (Advax) [24]. The addition of an adjuvant resulted in the induction of a broad range of antibodies.

Live attenuated SA14-14-2 was developed by a mutant virus with the deletion of the 3’ non-coding region and has been used mainly in China, inducing long-lasting effective neutralizing antibodies [25]. Besides inactivated and live attenuated JEV vaccines, recombinant yellow fever vaccine-based vectored vaccine is available. The 17D204 strain is a live attenuated yellow fever vaccine with sufficient immunogenicity and safety and has been used for a long time. *Yellow fever virus* is a family of *Flaviridae* family, like JEV, and the prM-E protein region was replaced by that of the SA14-14-2 live-attenuated JEV vaccine strain [12]. The 17D yellow fever vector has been applied to Dengue vaccines [26]. A live attenuated vaccine has been used as the back-bone for the development of recombinant vaccines, and the JEV prM-E gene was inserted into vaccinia virus cDNA [27].

In the present study, a live attenuated measles AIK-C strain was used as a virus vector. The AIK-C strain was developed in our institute, established through small plaque cloning at 32.5°C [28]. It showed the unique characteristics of extremely low or no virus growth at 39°C, and a low incidence of febrile illness after immunization [29]. The molecular basis for this attenuation was investigated: Pro at the 439 amino acid position of the P protein was responsible for the *ts* phenotype, and Leu at the 278 position of the F protein was important for small plaque formation [21, 30]. The AIK-C strain has been used as a recommended vaccine without any serious adverse events in Japan since 1977, and is also used for young infants [31, 32, 33]. An AIK-C-based chimera recombinant virus expressing the preM-E protein of Japanese encephalitis virus is theoretically safe and immunogenic. In the present study, JEV NT antibodies developed one week after immunization, more promptly than JEV EIA or measles PA antibodies. The prM-E protein may have been produced in infected cells and released. The same findings were obtained when cotton rats were immunized with recombinant measles AIK-C strains expressing respiratory syncytial virus [13].

The target age of the recommended vaccine for measles is one year of age and just before entering a primary school, while that for the JEV vaccine is more than 6 months of age, preferably at three years of age. Two doses of primary immunization are recommended, a booster one year after the primary immunization, and additional vaccination at 10 years of age using inactivated JEV vaccine [34]. The target generations for vaccination of measles and JEV vaccines are similar and the recombinant measles vaccine expressing JEV prM-E has the clinical benefit of inducing both humoral and cellular immune responses of the live vectored vaccine and simplifying the immunization schedule, thereby reducing the number of immunizations.
